# Large Vessel Occlusion in Patients With Minor Ischemic Stroke in a Population-Based Study. The Dijon Stroke Registry

**DOI:** 10.3389/fneur.2021.796046

**Published:** 2022-01-14

**Authors:** Gauthier Duloquin, Valentin Crespy, Pauline Jakubina, Maurice Giroud, Catherine Vergely, Yannick Béjot

**Affiliations:** Dijon Stroke Registry, Department of Neurology, University Hospital of Dijon, EA7460, Pathophysiology and Epidemiology of Cardio-cerebrovascular Disease (PEC2), University of Burgundy, Dijon, France

**Keywords:** stroke, ischemic stroke, registry, epidemiology, minor stroke, large vessel occlusion, population-based studies

## Abstract

**Introduction:**

Strategy for the acute management of minor ischemic stroke (IS) with large vessel occlusion (LVO) is under debate, especially the benefits of mechanical thrombectomy. The frequency of minor IS with LVO among overall patients is not well established. This study aimed to assess the proportion of minor IS and to depict characteristics of patients according to the presence of LVO in a comprehensive population-based setting.

**Methods:**

Patients with acute IS were prospectively identified among residents of Dijon, France, using a population-based registry (2013–2017). All arterial imaging exams were reviewed to assess arterial occlusion. Minor stroke was defined as that with a National Institutes of Health Stroke Scale (NIHSS) score of <6. Proportion of patients with LVO was estimated in the minor IS population. The clinical presentation of patients was compared according to the presence of an LVO.

**Results:**

Nine hundred seventy-one patients were registered, including 582 (59.9%) patients with a minor IS. Of these patients, 23 (4.0%) had a LVO. Patients with minor IS and LVO had more severe presentation [median 3 (IQR 2–5) vs. 2 (IQR 1–3), *p* = 0.001] with decreased consciousness (13.0 vs. 1.6%, *p*<0.001) and cortical signs (56.5 vs. 30.8%, *p* = 0.009), especially aphasia (34.8 vs. 15.4%, *p* = 0.013) and altered item level of consciousness (LOC) questions (26.1 vs. 11.6%, *p* = 0.037). In multivariable analyses, only NIHSS score (OR = 1.45 per point; 95% CI: 1.11–1.91, *p* = 0.007) was associated with proximal LVO in patients with minor IS.

**Conclusion:**

Large vessel occlusion (LVO) in minor stroke is non-exceptional, and our findings highlight the need for emergency arterial imaging in any patients suspected of acute stroke, including those with minor symptoms because of the absence of obvious predictors of proximal LVO.

## Introduction

The best strategy for the acute management of minor ischemic stroke (IS) is currently under debate. Indeed, in some cases, patients with minor symptoms at initial presentation of IS, defined as a National Institute of Health Stroke Scale (NIHSS) score of <5, may have a large vessel occlusion (LVO). In such a situation, a strong collateral circulation is usually associated with a relatively preserved cerebral blood perfusion, but there is a subsequent risk of early neurological deterioration when the adaptive process is overtaken if patients are not recanalized (1). Recent guidelines from the European Stroke Organization (ESO) recommended to administer intravenous thrombolysis (IVT) with recombinant tissue-type plasminogen activator (rt-PA) in patients with minor and disabling IS <4.5 h duration (2), in accordance with the results of a meta-analysis of randomized clinical trials showing the effectiveness of rt-PA on the outcome of these patients (3). In addition, the benefits of mechanical thrombectomy (MT) in patients with minor IS and LVO is currently evaluated in dedicated clinical trials (4). However, there is no standardized definition of minor “disabling” stroke, and the evaluation relies on judgement of physicians in clinical practice. Moreover, the frequency of minor IS with LVO among overall patients is not well known.

Therefore, the aim of this study was to assess the proportion of minor IS and to depict characteristics of patients according to the presence of LVO, in a comprehensive population-based setting.

## Methods

### Study Population and Case-Ascertainment Procedures

Data were obtained from the Dijon Stroke Registry (5–7), an ongoing prospective population-based study that complies with the criteria for conducting ideal incidence stroke studies (8), and the guidelines for the reporting of incidence and prevalence studies in neuroepidemiology according to Standards of Reporting of Neurological Disorders (9). The methodology of the Dijon Stroke Registry has been described extensively elsewhere (5–7). Briefly, case collection relies on multiple overlapping sources of information to identify hospitalized and not hospitalized cases of stroke among residents of the city of Dijon, France (156,000 inhabitants), including a review of medical records of all patients referred to the Dijon University Hospital where the only stroke unit in the country is located, a review of computerized hospital diagnostic codes using the International Classification of Diseases, Tenth Revision (ICD-10; I61; I62; I63; I64; G45; G46, and G81), a review of medical records from the departments of the private hospitals of the city and its suburbs, a cooperation with local general practitioners and private neurologists to identify stroke patients from home or nursing homes and Dijon residents who had a stroke when outside the city, a review of the medical records of patients identified from a computer-generated list of all requests for imaging to radiology centers in Dijon, and regular reviewing of death certificates to identify fatal strokes that occurred outside the hospital. The final adjudication of cases is systematically made by senior neurologists trained in stroke ascertainment according to the WHO diagnostic criteria (i.e., Rapidly developing clinical signs of focal, at time global, disturbance of cerebral function, lasting >24 h or leading to death with no apparent cause other than that vascular origin) (10).

For this study, analyses were restricted to patients with an acute IS between January 1, 2013 and December 31, 2017 and in whom data about arterial imaging (intracranial computed tomography angiography or magnetic resonance imaging) were available. The etiological classification of patients with IS was derived from the Trial of ORG 10172 in Acute Stroke Treatment (TOAST) classification (11) as follows: large artery atheroma, cardioembolic IS, lacunar IS due to small vessels disease, IS from other identified cause, IS from undetermined cause, and IS from multiple possible causes. The classification was made by a stroke-trained neurologist investigator of the Dijon Stroke Registry based on medical records, including complementary exams performed during the diagnostic workup of IS.

### Data Collection

As previously described, vascular risk factors, past medical history, and pre-stroke treatments were collected (7). Pre-stroke cognitive function (no cognitive impairment, mild cognitive impairment, and dementia) and functional status based on the premorbid modified Rankin Scale score were assessed. Pre-existing dependency was defined by a premorbid Rankin Scale score of >2. Stroke severity at onset was quantified using the NIHSS score obtained at the first clinical examination. Minor Stroke was defined as a NIHSS score of <6.

All cervical and intracranial arterial imaging exams were systematically reviewed by stroke-trained investigators to assess the presence and location of arterial occlusion responsible for the acute IS. A proximal LVO was defined as an occlusion site affecting the terminal intracranial internal carotid artery, M1 and M2 segments of the middle cerebral artery (including tandem occlusions), or A1 and A2 segment of the anterior cerebral artery, or the basilar artery. Patients with isolated extracranial internal carotid artery occlusion were not included in this group. In patients with minor IS and proximal occlusion, brain perfusion imaging including CT or MRI were reviewed when performed to assess the presence of a reduced cerebral perfusion in the territory of the occluded artery.

### Statistical Analyses

Proportions and mean values of baseline characteristics were compared between groups (patients with minor stroke vs. patients with non-minor stroke; minor stroke patients with vs. without proximal LVO) using the Chi-2 test and the Mann-Withney test. A multivariate logistic regression analysis was performed to evaluate factors associated with minor stroke. In models, we introduced age, sex, and variables with a *p* < 0.20 in unadjusted models. Another multivariate logistic regression analysis was performed to evaluate factors associated with proximal LVO among patients with minor IS. In models, variables with a *p* < 0.20 were introduced. Statistical analysis was performed with STATA 13 software (StataCorp LP, College Station, TX)

### Ethics

The Dijon Stroke Registry was approved using the following national ethics boards: The Comité d'Evaluation des Registres (French National Committee of Registers), Santé Publique France (French Institute for Public Health Surveillance), and the Commission Nationale Informatique et Liberté (French data protection authority). In accordance with the French legislation boards, the need for written patient consent was waived.

## Results

From January 1, 2013 to December 31, 2017, among the 1,060 recorded IS patients, 989 cases had available arterial imaging. In detail, 836 patients had a CT angiography, 456 had an MRI, and 683 patients had a US Doppler of cervical arteries, among whom 453 had a transcranial Doppler. The NIHSS score was available in 971 patients.

Among these patients, 582 (59.9%) suffered a minor stroke. Compared with non-minor stroke, minor stroke patients were younger (median age 78 vs. 82 years old, *p* < 0.001), had less frequent hypertension (69.2 vs. 76%, *p* = 0.02), atrial fibrillation (22.2 vs. 40.8%, *p* < 0.01), and history of coronary disease (13.0 vs. 18.1%, *p* = 0.03) (Table 1). In addition, pre-existing mild cognitive impairment (MCI) (10.2 vs. 16.4%) and dementia (9.5 vs. 18.2%) were less frequently observed in patients with minor stroke (*p* < 0.001) who were also less frequently functionally dependent (21.0 vs. 31.8%, *p* < 0.001) or institutionalized (7.1 vs. 16.0%, *p* < 0.001) before their stroke. IS etiology differed between patients with or without minor-stroke, with a greater proportion of cardioembolic IS observed among patients with non-minor stroke (45 vs. 24.6%). In multivariable analyses, past myocardial infarction (OR = 0.62; 95% CI:0.40−0.97, *p* = 0.035), MCI (OR = 0.58; 95% CI:0.36–0.95, *p* = 0.029), small vessel disease etiology (OR = 3.69; 95% CI: 1.76–7.74, *p* = 0.001), undetermined etiology (OR = 2.49; 95% CI: 1.52–4.06, *p* < 0.001), and IS with multiple causes (OR = 5.50; 95% CI: 1.97–15.34, *p* = 0.001) were associated with minor stroke.

**Table 1:** Characteristics of patients with minor (*n* = 582) vs. non-minor ischemic stroke (IS).

	**Patients with minor stroke** **(***N*** = 582)**	**Patients with non-minor stroke** **(***N*** = 389)**	***p*** **value**
Age, mean ± SD, y	73.2 (16.3)	78.5 (14.7)	
Age, median (IQR), y	78 (64–86)	82 (69–90)	<0.001
Male, *n* (%)	289 (49.6)	168 (43.2)	0.048
Hypertension, *n* (%)	403 (69.2)	295 (76.0)	0.021
Diabetes mellitus, *n* (%)	118 (20.3)	78 (20.1)	0.937
Hypercholesterolemia, *n* (%)	212 (36.6)	139 (35.8)	0.802
Current smoking, *n* (%)			0.125
No	449 (77.1)	292 (75.1)	
Yes	88 (15.1)	42 (10.8)	
Unknown	45 (7.7)	55 (14.1)	
History of AF, *n* (%)	129 (22.2)	158 (40.8)	<0.001
Current alcohol consumption, *n* (%)	33 (6.0)	18 (5.2)	0.614
Coronary heart disease, *n* (%)	75 (13.0)	70 (18.1)	0.031
Chronic heart failure, *n* (%)	40 (7.0)	39 (10.1)	0.084
Peripheral artery disease, *n* (%)	40 (6.9)	28 (7.2)	0.857
Active cancer, *n* (%)	20 (3.5)	19 (5.1)	0.229
Previous TIA, *n* (%)	85 (14.6)	41 (10.5)	0.065
Previous stroke, *n* (%)	117 (20.1)	89 (22.9)	0.306
Prestroke treatments, n(%)			
Antiplatelets agents	193 (33.4)	135 (35.3)	0.552
Anticoagulants	87 (1.1)	69 (18.0)	0.223
Antihypertensive treatment	365 (63.2)	258 (67.4)	0.180
Statins	144 (24.9)	80 (20.9)	0.148
Antidiabetic treatment	102 (17.7)	62 (16.2)	0.556
NIHSS score at onset, median (IQR)	2 (1 – 3)	13 (8 – 19)	<0.001
Prestroke cognitive status, *n* (%)			<0.001
No cognitive impairment	497 (80.4)	252 (65.5)	
MCI	59 (10.2)	63 (16.4)	
Dementia	55 (9.5)	70 (18.2)	
Premorbid mRS score>2, *n* (%)	122 (21.0)	123 (31.8)	<0.001
Living in an institution, *n* (%)	41 (7.1)	62 (16.0)	<0.001
TOAST classification, *n* (%)			<0.001
LAA	61 (10.5)	51 (13.1)	
CE	143 (24.6)	175 (45.0)	
SVD	52 (8.9)	14 (3.6)	
Other	43 (7.4)	25 (6.4)	
Undetermined	255 (43.8)	114 (29.3)	
Multiples causes	28 (4.)	10 (2.6)	
Acute revascularisation therapy			
IV thrombolysis only	36 (6.2)	70 (18.0)	
Mechanical thrombectomy only	4 (0.7)	38 (9.8)	
Combined treatment	3 (0.5)	17 (4.4)	

*AF, indicates atrial fibrillation; CE, cardioembolic; IQR, interquartile range; IV, intravenous; LAA, large artery atheroma; MCI, mild cognitive impairment; mRS, modified Rankin Scale; NIHSS, National Institutes of Health Stroke Scale; SVD, Small Vessel Disease; TIA, transient ischemic attack; and TOAST, Trial of ORG 10172 in Acute Stroke Treatment*.

**Figure 1:**
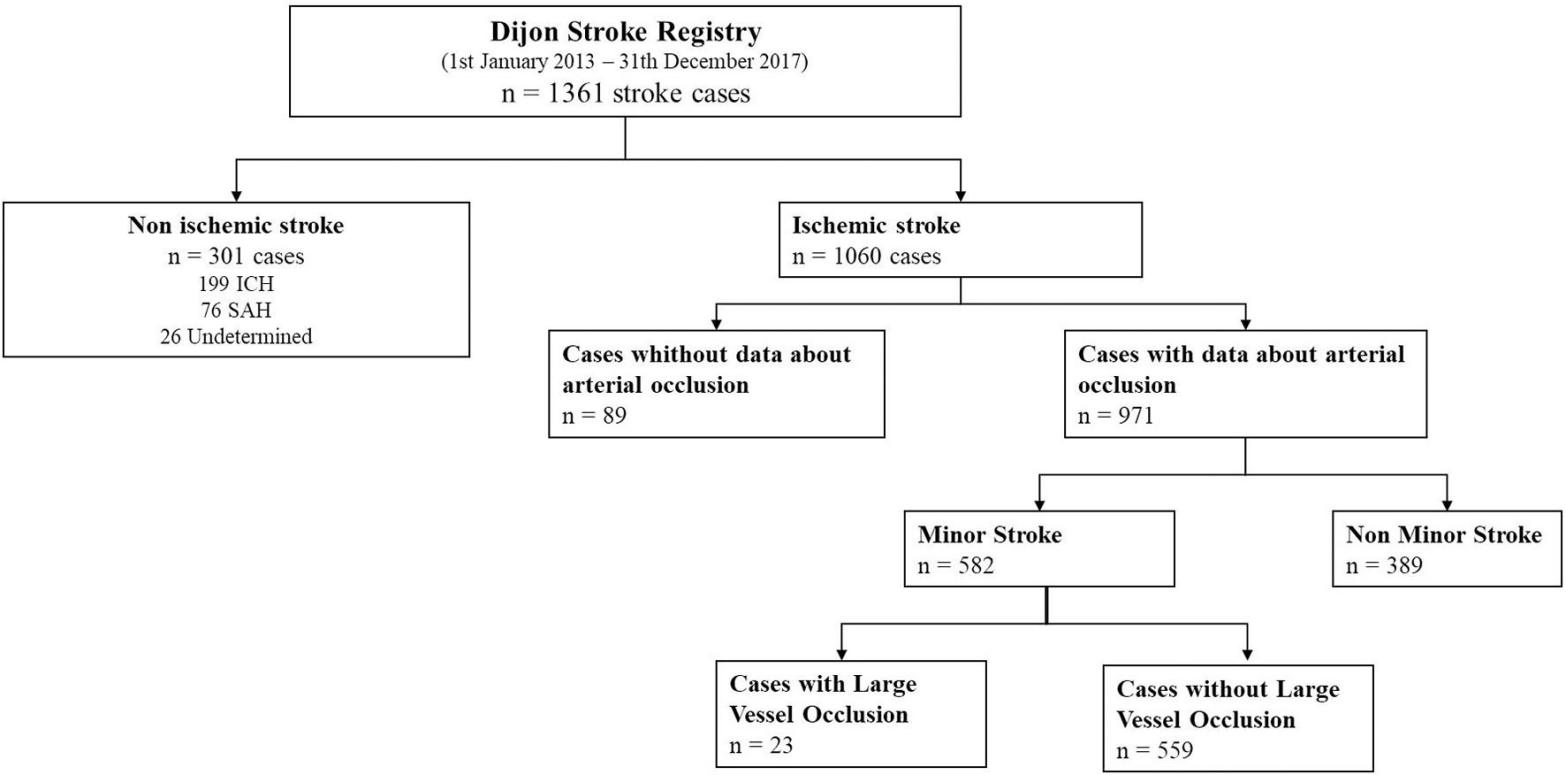
Study flow chart.

A total of 174 cases of IS with a proximal LVO were recorded in our study population. Among the 389 patients with non-minor stroke, 149 (38.3%) had a proximal LVO. In contrast, among the 582 patients with minor stroke, 23 (4.0%) had a proximal LVO (Figure 1). In these patients, the M1 or M2 segment of the MCA was occluded in 6 and 14 cases, respectively, whereas in 3 cases, the site of occlusion was the basilar artery. Three patients had an NIHSS score of 0, one patient scored 1, four patients scored 2, five patients scored 3, three patients scored 4, and seven patients scored 5. Among the 20 patients with a proximal occlusion of the MCA, 13 had perfusion imaging with an onset-to-imaging time ranging from 36 at 330 min. In all cases, perfusion imaging showed a hypoperfusion corresponding to the territory of the occluded artery. Ten patients received acute recanalization therapy (7 IV thrombolysis, 1 mechanical thrombectomy, and 2 bridging therapy). Among the 13 patients who did not receive acute recanalization therapy, three had a clinical deterioration with an increase in NIHSS score of 4 points per patient, among whom 2 had beneficiated from a brain perfusion imaging.

In patients with minor IS, those with a proximal LVO more often had atrial fibrillation (43.5 vs. 21.4%, *p* = 0.012), a higher NIHSS score [median 3 (IQR 2–5) vs. 2 (IQR 1–3), *p* = 0.001], and a greater proportion of cardioembolic IS mechanism (Table 2). In multivariable analyses, only NIHSS score (OR = 1.45 per point; 95% CI: 1.11–1.91, *p* = 0.007) was associated with proximal LVO in patients with minor stroke.

**Table 2:** Characteristics of minor ischemic stroke patients presenting with (*n* = 23) or without large vessel occlusion (LVO; *n* = 557).

	**Patients without LVO** **(***N*** = 559)**	**Patients with LVO** **(***N*** = 23)**	**P Value**
Age mean ± SD, y	73.4 ± 16.3	70.1 ± 17.8	0.287
Age median (IQR), y	78 (64–86)	72 (5–84)	
Male, *n* (%)	277 (49.6)	12 (52.2)	0.805
Hypertension, *n* (%)	389 (69.6)	14 (60.9)	0.375
Diabetes mellitus, *n* (%)	115 (20.6)	3 (13.0)	0.377
Hypercholesterolemia, *n* (%)	208 (37.4)	4 (17.4)	0.051
Current smoking, *n* (%)			0.361
Yes	85 (15.3)	3 (12.0)	
No	430 (76.8)	19 (84.0)	
Unknown	44 (7.9)	1 (4.0)	
History of AF, *n* (%)	119 (21.4)	10 (43.5)	0.012
Current alcohol consumption, *n* (%)	33 (6.3)	0 (0.0)	0.226
Coronary heart disease, *n* (%)	72 (13.0)	3 (13.0)	0.997
Chronic heart failure, *n* (%)	40 (7.3)	0 (0.0)	0.179
Peripheral artery disease, *n* (%)	39 (7.0)	1 (4.4)	0.618
Active cancer, *n* (%)	18 (3.3)	2 (8.7)	0.168
Previous TIA, *n* (%)	80 (14.3)	5 (21.7)	0.323
Previous stroke, *n* (%)	111 (19.9)	6 (27.3)	0.395
Prestroke treatments, *n* (%)	
Antiplatelets agents	185 (33.3)	8 (34.8)	0.885
Anticoagulants	84 (15.1)	3 (13.0)	0.783
Antihypertensive treatment	353 (63.6)	12 (52.2)	0.266
Statins	142 (25.6)	2 (8.7)	0.066
NIHSS score at onset, median (IQR)	2 (1–3)	3 (2–5)	0.001
Prestroke cognitive status, *n* (%)			0.567
No cognitive impairment	448 (80.3)	19 (82.6)	
MCI	58 (10.4)	1 (4.4)	
Dementia	52 (9.3)	3 (13.0)	
Premorbid mRS score>2, *n* (%)	117 (20.9)	5 (21.7)	0.926
Living in an institution, *n* (%)	41 (7.4)	0 (0.0)	0.177
TOAST classification, *n* (%)			0.011
LAA	60 (10.7)	1 (4.4)	
CE	130 (23.3)	13 (56.5)	
SVD	52 (9.3)	0 (0.0)	
Other	42 (7.5)	1 (4.4)	
Undetermined	247 (44.2)	8 (34.8)	
Multiple causes	28 (5.0)	0 (0.0)	

*AF, indicates atrial fibrillation; CE, cardioembolic; IQR, interquartile range; IV, intravenous; LAA, large artery atheroma; MCI, mild cognitive impairment; mRS, modified Rankin Scale; NIHSS, National Institutes of Health Stroke Scale; SVD, Small Vessel Disease; TIA, Transient Ischemic Attack; and TOAST, Trial of ORG 10172 in Acute Stroke Treatment*.

**Figure 2:**
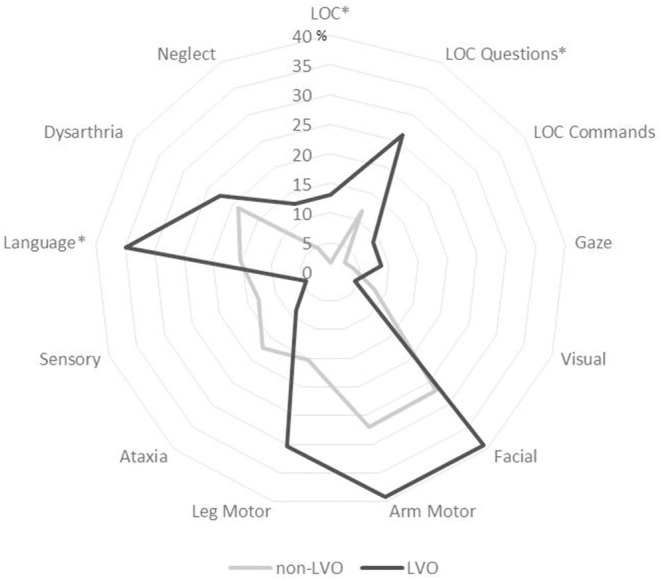
Proportion of patients with minor ischemic stroke who have impairment in each item of National Institutes of Health Stroke Scale NIHSS score according to the presence of proximal large vessel occlusion (LVO). *p < 0.05.

By studying the different items of the NIHSS, patients with minor IS and proximal LVO more often had decreased consciousness (13 vs. 1.6%, *p* < 0.001) and more often had cortical signs (56.5 vs. 30.8%, *p* = 0.009), especially aphasia (34.8 vs. 15.4%, *p* = 0.013) and altered item level of consciousness (LOC) Questions (26.1 vs. 11.6%, *p* = 0.037) (Figure 2).

In a sensitivity analysis, 441 patients (45.4%) had a minor IS, defined as that with an NIHSS score of ≤ 3. Among these patients, 13 (2.9%) had a proximal LVO. Of note, 3 out of 110 patients with a NIHSS score of 0 had a proximal LVO.

## Discussion

This study provided original data about the prevalence of proximal LVO in patients presenting with minor IS in a large population-based setting. We observed that ~4% of patients with a mild clinical presentation had an LVO, thus representing those potentially eligible for mechanical thrombectomy. Although LVO in patients with minor IS was more frequently noticed in patients with atrial fibrillation and/or a cardioembolic etiology, only a greater NIHSS score was independently associated with LVO, which was explained by more frequent decreased consciousness, aphasia, or altered item LOCquestions.

Different definitions are used to define the minor stroke in the literature. Fischer et al. suggested several definitions of minor stroke and concluded that a maximum score of 1 on every baseline NIHSS score, except level consciousness items, or a total NIHSS score of ≤3 could be the best definition (12). In the Oxford Vascular Study (OXVASC), a NIHSS score of <3 was used to define minor stroke (13). Regarding recent randomized clinical trials focusing on dual antiplatelet therapy in secondary prevention of IS patients, both Platelet-oriented Inhibition in New TIA and Minor Ischemic Stroke (POINT) (14) and Clopidogrel in High-risk Patients with Acute Non-disabling Cerebrovascular Events (CHANCE) (15) trials considered minor stroke if the NIHSS score was ≤ 3. Conversely, in Acute Stroke Or Transient IsChaemic Attack TReated With Aspirin or Ticagrelor and Patient OutcomES (SOCRATES) (16) and The Acute Stroke or Transient Ischaemic Attack treated with Tricagelor and Tricagelor and Acetylsalicylic Acid for Prevention of Stroke and Death (THALES) (17) trials, minor stroke was defined as that with a NIHSS score of ≤ 5. Some authors suggested a definition of mild severity as a NIHSS score <5 (18, 19). Recent guidelines from the ESO on the use of dual antiplatelets therapy in minor IS used a NIHSS score ≤ 3 as a threshold (20, 21). However, for the management of IS with endovascular therapy, current recommendations used a NIHSS score of <6 for defining IS with mild symptoms (22, 23). Whether these patients should benefit from mechanical thrombectomy is a challenging issue. Therefore, we used this definition to assess the true prevalence of LVO in minor IS.

Minor IS, defined as a NIHSS score of <6, accounted for ~60% of overall IS patients in our population. So as to compare with the Oxford Vascular (OXVASC) study, we found a similar proportion of minor IS when considering a definition with a NIHSS score <3 (45% in our study vs. 47% in the Oxford Vascular Study). This high rate of minor IS reflects the fact that, in both studies, we used a population-based setting rather than a hospital-based recruitment that would have led to higher clinical severity of included patients (24).

Our study provides new information on the prevalence of LVO in patients with minor IS. Although this prevalence was relatively low (4%), we did not find any factor associated with the presence of LVO in these patients, except the NIHSS score. However, the difference was very small, and, therefore, it is not useful for the discrimination between patients with vs. without occlusion. Consequently, our findings suggest that in a patient presenting with a clinical picture of minor stroke, it is impossible to easily predict the existence of a proximal LVO. This is important in the current context of discussion about the best therapeutic strategy in this patient, i.e., whether to administer IV thrombolysis and/or mechanical thrombectomy, and it should be considered that until proven otherwise, these patients may have a LVO and may therefore benefit from urgent brain and arterial imaging even if the neurological symptoms are mild. In addition, we noticed that all patients with minor IS and a proximal LVO of the anterior circulation had a hypoperfusion in the corresponding arterial territory when perfusion imaging was performed. A majority of these patients received IV thrombolysis despite the fact that the guidelines regarding the indication of this therapy were not established at the time this study was conducted. Of note, 2 out of 3 patients with proximal occlusion and a hypoperfusion and who did not receive IV thrombolysis had an early neurological deterioration. This suggests that brain perfusion imaging could be useful for the selection of minor IS patients eligible to acute revascularization therapy.

The major strength of our study is the use of a population-based registry and a relatively large sample size of patients. The reliability of the classification of patients as having or not having an LVO was ensured by a systematic review of all arterial imaging exams by stroke-trained investigators. However, our study was limited by a small number of cases with LVO, thus limiting the study power and additional subgroup analyses.

To conclude, LVO in minor stroke is non-exceptional, and our findings highlight the need for emergency arterial imaging in any patients suspected of acute stroke, including those with minor symptoms, because of the absence of obvious predictors of proximal LVO.

## Data Availability Statement

The datasets presented in this article are not readily available because of restrictions due to national legislation. Requests to access the datasets should be directed to yannick.bejot@chu-dijon.fr.

## Author Contributions

GD: study concept and design, acquisition, analysis and interpretation of data, reviewing arterial imaging, and drafting and revising the manuscript for content. VC, PJ, MG, and CV: acquisition of data and critical revision of manuscript for intellectual content. YB: study concept and design, acquisition, analysis and interpretation of data, study supervision, obtaining funding, and drafting and revising the manuscript for content. All authors contributed to the article and approved the submitted version.

## Funding

The Dijon Stroke Registry was supported by Santé Publique France, Institut national de la santé et de la recherche médicale (INSERM), and Dijon University Hospital.

## Conflict of Interest

YB reports personal fees from BMS, Pfizer, Medtronic, Amgen, Servier, NovoNordisk, and Boehringer-Ingelheim, outside the submitted work. The remaining authors declare that the research was conducted in the absence of any commercial or financial relationships that could be construed as a potential conflict of interest.

## Publisher's Note

All claims expressed in this article are solely those of the authors and do not necessarily represent those of their affiliated organizations, or those of the publisher, the editors and the reviewers. Any product that may be evaluated in this article, or claim that may be made by its manufacturer, is not guaranteed or endorsed by the publisher.
